# Exogenous carbon monoxide suppresses adaptive response induced in zebrafish embryos *in vivo* by microbeam protons

**DOI:** 10.1093/jrr/rrt165

**Published:** 2014-03

**Authors:** Viann Wing Yan Choi, Candy Yuen Ping Ng, Alisa Kobayashi, Teruaki Konishi, Masakazu Oikawa, Shuk Han Cheng, Peter Kwan Ngok Yu

**Affiliations:** 1 Department of Physics and Materials Science, City University of Hong Kong, Hong Kong; 2 Department of Technical Support and Development, National Institute of Radiological Sciences, 4-9-1 Anagawa, Inage, Chiba 263-8555, Japan; 3 Department of Biology and Chemistry, City University of Hong Kong, Hong Kong; 4 State Key Laboratory in Marine Pollution, City University of Hong Kong, Hong Kong

**Keywords:** protons, adaptive response, zebrafish embryos

## Abstract

Dechorionated embryos of the zebrafish, *Danio rerio*, irradiated at 5 h post-fertilization (hpf) with 30 protons delivered to 10 separate positions each with an energy of 3.4 MeV from the microbeam irradiation facility (Single-Particle Irradiation System to Cell, acronym as SPICE) at the National Institute of Radiological Sciences (NIRS), developed radioadaptive response (RAR) against a subsequent challenging exposure of 2 Gy of X-ray irradiation at 10 hpf, corroborated by reduced apoptotic signals at 25 hpf revealed through terminal dUTP transferase-mediated nick end-labeling assay.

The effects of the CO liberator tricarbonylchloro(glycinato)ruthenium (II) (CORM-3) on the induction of RAR were examined by transferring the irradiated embryos to freshly prepared medium with the chemical at different time points after the application of the priming dose. Our results showed that transfer of irradiated embryos into media with CORM-3 at 0, 1, 2 and 3 h after application of priming exposure significantly suppressed RAR, while transfer at 5 h did not suppress RAR. This was attributed to the protection of bystander cells from the released CO, which caused less *de novo* synthesis of factors and thus less efficient induction of RAR. Once the factors were synthesized, RAR was induced, which would not be further affected by the application of CORM-3 introduced at 5 h after the application of the priming dose.

Clinical Trial Registration number if required: None.

